# Chronic Spontaneous Urticaria Triggered by the AstraZeneca/Oxford COVID-19 Vaccine with Achieved Remission: A Case Report

**DOI:** 10.1177/21526567211068458

**Published:** 2022-01-10

**Authors:** Stephanie G Brooks, Anna M De Jong, Mina Abbaslou, Gordon Sussman

**Affiliations:** 1 12366 Temerty Faculty of Medicine, University of Toronto, Toronto, Ontario, Canada; 2 Gordon Sussman Clinical Research, Inc., Toronto, Ontario, Canada; 3 6221 University of Western Ontario, London, Ontario, Canada; 4 12366 Department of Medicine and Division of Clinical Immunology & Allergy, University of Toronto, Toronto, Ontario, Canada

**Keywords:** urticaria, COVID-19, vaccine reaction

## Abstract

New adverse reactions to the COVID-19 vaccines are being identified as vaccination rates increase worldwide. Recently, there have been two reports of Moderna (mRNA-1273) vaccine induced relapse of chronic spontaneous urticaria (CSU) that was previously well controlled. Herein, we report a case of AstraZeneca/Oxford (ChAdOx1) vaccine triggered CSU in a patient with no history of CSU with achieved remission.

Acute urticaria is a common adverse reaction following the AstraZeneca/Oxford (ChAdOx1), Pfizer/BioNTech (BNT162b2) and Moderna (mRNA-1273) COVID-19 vaccines.^1–3^ Chronic urticaria, defined as urticaria persisting beyond six weeks, triggered by the Moderna mRNA vaccine was recently reported.^4^ We present a unique case of COVID-19 vaccine induced chronic spontaneous urticaria (CSU) triggered by the AstraZeneca/Oxford COVID-19 vaccine in a patient with no history of CSU. Written patient informed consent was received.

A 60-year-old male with environmental allergies and a history of asthma received the first dose of the AstraZeneca/Oxford COVID-19 vaccine on March 15, 2021. The patient had no symptoms until five days later, when he developed erythema on his palms and cheeks followed by a pruritic rash on his scalp, face, neck, shoulders and armpits. He denied use of non-steroidal anti-inflammatory drugs, antibiotics or changes in diet during this time. He visited a pharmacist who recommended bilastine 20 mg daily, an antihistamine he is currently prescribed for his environmental allergies. On April 2, the patient saw his family doctor who continued treatment with bilastine and prescribed betamethasone and hydrocortisone creams without success. Fourteen days later he was given a five-day course of prednisone, which resolved his symptoms; however, he developed a generalized pruritic rash that would wax and wane as well as angioedema immediately upon discontinuation. To manage his symptoms, he took frequent cold showers and continued with bilastine. On May 4, the patient saw a dermatologist who switched him to rupatadine 20 mg, prescribed hydroxyzine 25 mg for sleep, and re-treated him with a 15-day course of prednisone followed by a taper. A mild rash returned upon discontinuation, but he eventually achieved remission in June 2021.

The patient was seen in an allergy and immunology clinic on June 23, 2021. Images of his rash showed red wheals, characteristic of urticaria. His bloodwork was within normal range, including immunoglobulins. Protein electrophoresis and immunofixation did not suggest a monoclonal pattern. There was no history of reaction to vaccines or laxatives containing polyethylene glycol. The patient reported a potential anaphylactic reaction to immunotherapy for his environmental allergies over 30 years ago; however, this is a known side effect. He did not endorse elevated stress levels associated with receiving the vaccine. The patient was diagnosed with CSU that appeared to be triggered by the AstraZeneca/Oxford COVID-19 vaccine as no other precipitants were identified and was advised to receive an mRNA vaccine for his second dose. On July 2, 2021, the patient received the Pfizer/BioNTech vaccine. He pre-medicated with bilastine 20 mg. An hour post vaccination, the patient developed pruritus of the palms, as well as a similar rash on his scalp and neck. However, these symptoms resolved five days later with continuation of bilastine 20 mg and have not returned.

New adverse reactions to the COVID-19 vaccines are being identified as vaccination rates increase worldwide. Cutaneous reactions are the most frequently reported, in keeping with the types of adverse reactions seen with more established vaccines, such as the MMR and Varicella vaccines.^3,5^ The association of CSU and the AstraZeneca/Oxford vaccine in this report warrants further investigation. The COVID-19 pandemic has imposed numerous challenges, and the possibility of stress-induced CSU in this patient cannot be completely dismissed despite his denial. Public access to the AstraZeneca/Oxford vaccine for immunological testing may enable confirmatory testing of vaccine-induced reactions. However, in the meantime, patients with similar symptoms should be referred to an allergist and/or immunologist specialist for appropriate management.
